# Plumbagin resurrect colistin susceptible against colistin-resistant *Pseudomonas aeruginosa in vitro* and *in vivo*

**DOI:** 10.3389/fmicb.2022.1020652

**Published:** 2022-09-29

**Authors:** Yue Wang, Jingchun Kong, Xiaodong Zhang, Yan Liu, Zeyu Huang, Lu Yuan, Ying Zhang, Jianming Cao, Lijiang Chen, Yong Liu, Tieli Zhou

**Affiliations:** ^1^Department of Clinical Laboratory, The First Affiliated Hospital of Wenzhou Medical University, Wenzhou, Zhejiang, China; ^2^Department of Medical Lab Science, School of Laboratory Medicine and Life Science, Wenzhou Medical University, Wenzhou, Zhejiang, China; ^3^Oujiang Laboratory (Zhejiang Lab for Regenerative Medicine, Vision and Brain Health), Wenzhou Institute, University of Chinese Academy of Sciences, Wenzhou, Zhejiang, China

**Keywords:** *Pseudomonas aeruginosa*, colistin-resistant, plumbagin, biofilm, synergy effect

## Abstract

The global emergence and spread of multi-drug resistant (MDR) strains is becoming increasingly worrisome due to the overuse of broad-spectrum antibiotics. Colistin, the last resort for treating MDR strains infections, has once again returned to the clinician’s choice. However, with the widespread use of colistin, colistin-resistant gram-negative bacteria (GNB) have subsequently emerged, including colistin-resistant *Pseudomonas aeruginosa* (COL-R PA). Therefore, available solutions are urgently needed to respond to this situation. Here, we inspiringly found that the combination of plumbagin and colistin had an efficiently inhibitory effect for colistin-resistant *P. aeruginosa in vitro* through checkerboard assay and time-kill assay. The combinatorial inhibition of biofilm formation was clearly demonstrated by crystal violet staining and scanning electron microscopy (SEM), and this combination can not only inhibited biofilm formation but also eradicated the mature biofilm. Erythrocytes hemolysis test showed that plumbagin has negligible hemolysis ability. In addition, the increased survival rate of *Galleria mellonella* (*G. mellonella*) larva confirmed this combination as same as effective *in vivo*. As for the mechanism of this combination, propidium iodide (PI) staining showed colistin combined with plumbagin could significantly change the membrane permeability, thus exerting synergistic antibacterial activity. In conclusion, the combination of plumbagin and colistin shows a prominently synergistic antibacterial effect *in vitro* and *in vivo*, providing a promising option for the therapy of COL-R PA infection.

## Introduction

*Pseudomonas aeruginosa* (*P. aeruginosa*) is a Gram-negative non-fermentative bacillus and one of the main opportunistic pathogens leading to nosocomial acute and chronic infections (Shariati et al., 2018). The World Health Organization (WHO) identified *P. aeruginosa* as one of the most life-threatening bacteria in 2017 and designated it as a priority pathogen for new antibiotic research and development (Hazlett et al., 2019). Over the past few years, the widespread use and even abuse/misuse of antibiotics have resulted in the emergence of multi-drug resistant (MDR) strains (Son et al., 2019). However, the lack of novel antimicrobials available to treat MDR bacteria-induced infections is scary (Bjarnsholt, 2013; Kaye et al., 2016).

Besides, bacteria are prone to produce bacterial biofilms. A biofilm is produced by an organized population of bacteria enclosed by extracellular macromolecules attached to the surface of living or inanimate objects. Bacteria inside biofilm are highly resistant to antibiotics and the host immune defense mechanisms (Trejo-Hernández et al., 2014). They colonize surfaces in the human skin, wounds, and the lumen of organs, causing persistent infections such as chronic wound infections, endocarditis, keratitis, and implant-associated lung infections (Singh et al., 2000). Particularly, *P. aeruginosa* biofilms cause chronic lung infections in cystic fibrosis (CF) patients (Marczak et al., 2005). Therefore, treating *P. aeruginosa* biofilm-associated infections is challenging, especially for the infections induced by MDR *P. aeruginosa*.

Colistin (Polymyxin E), a cationic lipopeptide antibiotic, targets the lipid A of lipopolysaccharide (LPS) and disturbs the out membrane of Gram-negative bacteria (GNB). However, colistin has gradually been abandoned due to its potential nephrotoxicity and the emergence of other new drugs (Muñoz and Hergenrother, 2021). By the mid-1990s, the polymyxins had re-emerged as a last-resort treatment against MDR GNB, particularly *P. aeruginosa*, *Acinetobacter baumannii* (*A. baumannii*), *Klebsiella pneumoniae* (*K. pneumoniae*), and *Escherichia coli* (*E. coli*), which are resistant against all other available antibiotics (Mandler et al., 2018). Unfortunately, colistin-resistant strains have also emerged with the unreasonable use of colistin. MDR *P. aeruginosa*, particularly COL-R PA has been classified by the Centers for Disease Control and Prevention as one of the essential organisms posing a severe threat to human health worldwide (Mikhail et al., 2019).

Plumbagin (5-hydroxy-2-methyl-1, 4-naphthoquinone), is a quinone found in the plants of Droseraceae, Plumbaginaceae, Ancestrocladaceae, and Dioncophyllaceae families (Marczak et al., 2005). Plumbagin has shown its potential therapeutic benefits on numerous chronic diseases like breast cancer, non-small cell lung cancer, melanoma, ovarian, squamous cell carcinomas, pancreatic cancer, and prostate cancer (Hafeez et al., 2013). Also, plumbagin can regulate Gram-negative bacterial virulence and biofilm formation by inhibiting the quorum-sensing (QS) system (Qais et al., 2021). Therefore, plumbagin, as a component of Chinese herbal medicine, has a broad prospect in the medical treatment of many diseases. The synergy effect of gentamicin and plumbagin against carbapenem-resistant *K. pneumoniae* bacteria has been reported (Chen et al., 2020), but the antibacterial and anti-biofilm activities of plumbagin and colistin against COL-R PA is still unclear.

Therefore, we wonder whether the combination of plumbagin and colistin is effective against COL-R PA and, more importantly, against the biofilm of COL-R PA. In this study, we investigated the synergistic effect of plumbagin and colistin against COL-R PA *in vitro* and *in vivo*.

## Materials and methods

### Isolates and growth condition

A total of seven non-duplicate clinical COL-R PA strains isolated from the First Affiliated Hospital of Wenzhou Medical University were randomly selected. In accordance with the manufacturer’s instructions, MALDI-TOF-MS (BIOMERIQUE, France) was used to identify all isolates. Luria Bertani (LB) (Thermo Fisher Scientific, America) broth supplemented with 30% glycerol was used to freeze isolates (−80°C). The quality control strain *P. aeruginosa* ATCC 27853 was obtained from the National Center for Clinical Laboratory.

### Antibiotics and solvents preparation

Plumbagin was purchased from Meigu Biotechnology Co., Ltd. (Zhejiang, China). The other antibiotics used in this study were: aztreonam (ATM), ceftazidime/avibactam (CZA), cefepime (FEP), imipenem (IPM), meropenem (MEM), ciprofloxacin (CIP), levofloxacin (LVX), gentamicin (GEN), tobramycin (TOB), piperacillin (PIP) and colistin (COL) (Wenzhou Kangtai Biotechnology Co., Ltd., Zhejiang, China). Antimicrobial susceptibility testing and checkerboard assays were conducted using cationic adjusted Mueller-Hinton broth (CAMHB) (OXIOD, Thermo Fisher Scientific, British).

### Antimicrobials susceptibility testing

The minimum inhibitory concentrations (MICs) of antibiotics and plumbagin against the seven COL-R PA clinical isolates were determined using the micro-broth dilution method (Richter et al., 2017). Briefly, A series of diluted antibiotics in 96-well microtiter plates was prepared. Each well of the plates was inoculated with a 100 μl bacterial suspension at 37°C for 16–18 h. The breakpoint of antibiotics was interpreted accordingly to the latest Clinical and Laboratory Standards Institute (CLSI). The experiment was repeated three times independently.

### Checkerboard assays

The synergistic antibacterial effect of plumbagin and colistin was explored using the checkerboard assay, as described elsewhere, with slight modification (Gaibani et al., 2014). Briefly, colistin was used as drug A and double-diluted consecutively. Different concentrations of plumbagin were prepared and used as drug B. The bacterial suspension was diluted with CAMHB. Then, 100 μl bacterial suspension was added to 96-well microplates and mixed with drugs 100 μl A and 50 μl B. The resulting mixtures were incubated at 37°C for 16–18 h to observe the results. The synergistic effect of colistin combined with plumbagin was investigated using the fractional inhibitory concentration index (FICI). FICI was calculated using the formula: FICI = FIC_*A*_ + FIC_*B*_; FIC_*A*_ = MIC_*A*_ in combination/MIC_*A*_ alone; FIC_*B*_ = MIC_*B*_ in combination/MIC_*B*_ alone. The interactions were explained as follows: FICI ≤0.5, Synergistic effect; 0.5< FICI ≤4, No interaction; Antagonism of FICI >4 (She et al., 2021). All experiments were conducted in triplicate.

### Time-kill assay

To further determine the synergistic antibacterial effect of plumbagin and colistin, the time-kill assay was performed as previously described, with minor modifications (Berard et al., 2016; Zakuan and Suresh, 2018). Four groups: control group (without drug), colistin group, plumbagin group, and colistin/plumbagin combination group were set. The colistin group with a final concentration of 0.5–2 μg/ml, the plumbagin group with a final concentration of 32–64 μg/ml, and the combined group was added a monotherapy concentration of the corresponding isolates. 100 μl of 0.5 McFarland bacterial suspension was added to LB broth with different treatments, then cultured at 37°C for 180 rpm. Cultures were taken out at 0, 2, 4, 6, 12, and 24 h and spread onto the LB agar plates. Then plates were incubated at 37°C overnight for colony counting. Compared with either drug alone, bactericidal action is defined as a ≥ 3 log_10_ CFU/ml reduction, and synergistic activity was defined as a ≥ 2 log_10_ CFU/ml decline.

### Biofilm formation inhibition test

To investigate the effect of plumbagin in combination with colistin on the biofilm formation of COL-R PA, performed the same as described with minor modifications (Thibeaux et al., 2020). Single colony was adjusted to 0.5 McFarland, then the bacterial solution was diluted at 1: 100 with LB broth and added 100 μl to each well of 96-well plates. Colistin and plumbagin were also diluted to a specific concentration with LB broth, 100 μl for each drug and 50 μl for the combination. Next, after the 96-well plates were incubated at 37°C for 24 h, the bacterial solution in the well was poured out and washed three times with sterile PBS to remove planktonic bacteria. Then, the plates were inverted for natural drying, and 200 μl crystal violet was added and placed at 37°C for staining. After 15 min, the crystal violet was sucked out and washed with sterile PBS. Next, plates were dried naturally, and 200 μl of 95% ethanol plus 5% acetic acid was added to dissolve crystal violet. Biofilm biomass was quantitated through absorbance at 595 nm. All experiments were conducted in triplicate.

### Biofilm eradication test

The eradication of the preformed biofilm by combining plumbagin and colistin was explored, as described above, with minor modifications (Okamoto-Shibayama et al., 2021). The bacterial suspension was prepared the same as mentioned above, then added to 100 μl per well and incubated for 24 h at 37°C to form biofilm. Afterward, wells were aspirated and washed thrice with sterile PBS to remove planktonic bacteria, and the residual liquid was aspirated with blotting paper. The prepared colistin and plumbagin were added to each well and cultured at 37°C for 24 h. As previously described, stained with crystal violet, the absorbance at 595 nm was measured.

### Scanning electron microscope

Scanning electron microscope was used to further investigate biofilm’s inhibition by combining plumbagin/colistin with minor modifications (Sun et al., 2021). In the SEM experiment, TL2314 was selected as the experimental strain. We performed sterile cell culture experiments on 6-well plates (NEST Biotechnology, China). The four groups were set up: control group, colistin monotherapy group, plumbagin monotherapy group, and combination group. First, sterile coverslips (24 mm × 24 mm) were placed into six-well plates to provide a biofilm-forming surface, to which 100 μl of 0.5 McFarland bacterial suspension was added to six-well plates. Subsequently, 1,900 μl LB broth was added to the control group, LB broth with a final concentration of 1 μg/ml colistin or 16 μg/ml plumbagin was added to the monotherapy group. LB broth containing the final concentration of 1 μg/ml colistin and 16 μg/ml plumbagin was added to the combination group and incubated at 37°C for 18–24 h. The coverslips were washed thrice with PBS, fixed with 2.5% glutaraldehyde at 4°C for 4 h, and dehydrated with a series of gradient concentrations (30, 50, 70, 90, and 100%) of ethanol for 10 min. The final samples were dried at room temperature, sprayed with gold, and then observed by SEM (S-3000N, Japan).

### Hemolysis test

We further verified the effect of plumbagin on red blood cells (RBCs) as described elsewhere (Zhang et al., 2022). Briefly, fresh blood from healthy adults was collected and centrifuged to remove the plasma and mononuclear cells. The RBCs were washed thrice with sterile PBS and centrifuged at 3000 rpm for 5 min. The RBC suspension was diluted with normal saline so that it eventually became 5% RBC suspension. Various concentrations of 250 μl of plumbagin (8–128 μg/ml) were added to 250 μl of 5% RBC suspension. After incubation in the water bath at 37°C for 1 h, centrifugation at 4000 rpm for 5 min, the supernatant was absorbed into 96-well plates, and the absorbance at 545 nm was measured to reflect hemolysis rate. The negative control group was only added with sterile PBS, and the positive control group was added with 0.1% Triton X-100. The experiment was performed three times.

### *In vivo* synergy in the *Galleria mellonella* infection model

We evaluated the synergistic effects of plumbagin and colistin *in vivo* in the *G. mellonella* infection model with minor modifications (Tsai et al., 2016). Only the uniform and milky *G. mellonella* larvae weighing about 250–300 mg were selected for the experiment. TL2917 and TL2314 were used as experimental strains. In short, a single colony was selected and adjusted to 0.5 McFarland. The cultures of TL2917 and TL2314 were diluted to 1.5 × 10^3^ CFU/ml. The following four groups were established: control group, plumbagin monotherapy group (32 and 64 μg/ml × 7), colistin monotherapy group (1 and 2 μg/ml × 7), and combination treatment group. Ten microliter of bacterial suspension was injected into the left posterior front row of *G. mellonella* using a microinjector, after 2 h, 10 μl of drugs were injected into the right offside posterior front row. Only injected sterile saline was the negative control. Larvae were then placed at 37°C dim room to record a 7-day survival rate. The mortality of *G. mellonella* larvae was determined primarily by documentation of changes in skin color and non-response to stimuli. The Kaplan–Meier analysis and the log-rank test were adopted to analyze *G. mellonella*.

### Cell membrane permeability assay

The cell membrane permeability test was used to investigate the synergistic antibacterial mechanism of plumbagin and colistin (Qiu et al., 2021). TL2314 was selected as the experimental strain. Exponential phase bacteria were treated with colistin (0.5, 1, 2 μg/ml) or plumbagin (4, 8, 16, 32 μg/ml) alone and in combination (1 + 4, 1 + 8, 1 + 16, and 1 + 32 μg/ml) for 2 h and incubated with propidium iodide (PI, 50 μg/ml) for 30 min at 37°C. The fluorescence images were recorded on a fluorescence microscope (Nikon, Japan) at 561 nm.

### Statistical analysis

Data were expressed as mean ± standard deviation over at least three independent trials. Significance was determined by using One-way ANOVA and mentioned as *P*-value <0.05 (noted with*), *P*-value <0.01 (noted with**) and *P*-value <0.001 (noted with***). Statistical analyses were performed using Graph Pad Prism 8.3 statistical software.

## Results

### Most of the test isolates exhibit multi-drug resistant phenotypes

Table 1 shows the MICs of common antibiotics for colistin-resistant isolates of *P. aeruginosa*. MDR bacteria are mainly defined as bacteria that are resistant to three or more types of antibiotics in clinical use. MDR traits were found in almost all of these isolates. The MIC of the recorded colistin against all strains was 4–128 μg/ml. The MIC of plumbagin was not applicable for all strains except TL3008, indicating that plumbagin had no significant antibacterial activity against the tested isolates.

**TABLE 1:** The minimum inhibitory concentration (MIC) values of commonly clinical antibiotics and plumbagin against colistin-resistant clinical isolates.

Strains	Antibiotics	IPM	ATM	LVX	FEP	MEM	GEN	CIP	TOB	PIP	CZA	COL	PLU
	
	Breakpoints (S–R)	2–8	8–32	1–4	8–32	2–8	4–16	0.5–2	4–16	16–128	8–16	2–4	NA
TL1671	MIC (μg/ml)	2^S^	8^S^	1^S^	8^S^	0.5^S^	2^S^	0.25^S^	1^S^	8^S^	4^S^	8^R^	>256
TL1736		16^R^	8^S^	1^S^	2^S^	256^R^	32^R^	1^I^	8^I^	256^R^	2^S^	8^R^	>256
TL2314		4^I^	16^I^	2^S^	16^I^	4^I^	8^I^	0.5^S^	2^S^	256^R^	4^S^	4^R^	>256
TL2917		16^R^	32^R^	2^S^	16^I^	256^R^	8^I^	0.25^S^	8^I^	256^R^	8^I^	4^R^	>256
TL2967		16^R^	128^R^	16^R^	32^R^	128^R^	8^I^	8^R^	8^I^	256^R^	16^R^	4^R^	>256
TL3008		16^R^	4^S^	1^S^	4^S^	8^R^	16^R^	0.5^S^	4^S^	8^S^	4^S^	64^R^	128
TL3086		128^R^	128^R^	8^R^	16^I^	128^R^	128^R^	16^R^	128^R^	128^R^	4^S^	128^R^	>256

MIC, COL-R PA inhibitory concentration; IPM, imipenem; ATM, aztreonam; LVX, levofloxacin; FEP, cefepime; MEM, meropenem; GEN, gentamicin; CIP, ciprofloxacin; TOB, tobramycin; PIP, piperacillin; CZA, ceftazidime/avibactam; COL, colistin; S, Susceptible; I, intermediate; R, resistance. NA is not applicable.

### Plumbagin and colistin combination exhibits synergistic antimicrobial effects on colistin-resistant isolates of *Pseudomonas aeruginosa*

The synergistic effects of colistin in combination with plumbagin on CR-*P. aeruginosa* were evaluated. As shown in Table 2, the combination of plumbagin and colistin showed synergistic activity with FICI <0.5 for all strains. When colistin was used in combination with plumbagin, the MIC of colistin was significantly decreased (4–128 fold decrease), all strains’ susceptibility to colistin was significantly increased, and the resistant phenotype reverted to the susceptible phenotype.

**TABLE 2:** The minimum inhibitory concentration (MIC) and FICI values for colistin/plumbagin combination against colistin-resistant *Pseudomonas aeruginosa* isolates.

	MIC (μ g/ml)		FIC (μ g/ml)		FICI	Interpretation
				
Strains	colistin	plumbagin	colistin	plumbagin		
TL1671	8	>256	0.25	8	0.047	Synergy
TL1736	8	>256	2	2	0.254	Synergy
TL2314	4	>256	0.06	8	0.031	Synergy
TL2917	4	>256	0.5	8	0.141	Synergy
TL2967	4	>256	0.5	16	0.156	Synergy
TL3008	64	128	1	16	0.141	Synergy
TL3086	128	>256	1	8	0.023	Synergy

FICI, fractional inhibitory concentration index.

### Time-kill curve assay shows the synergistic antimicrobial function of plumbagin in combination with colistin

**FIGURE 1:**
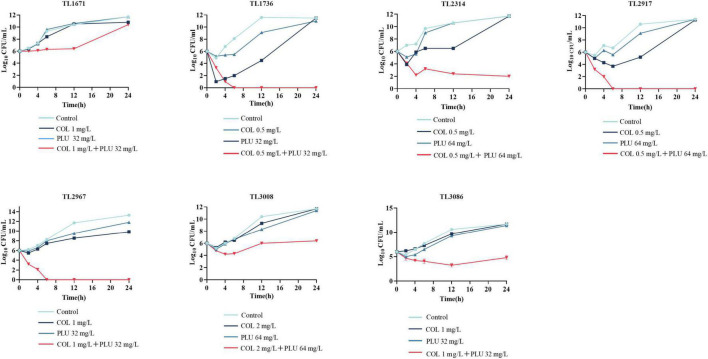
Time-kill curve diagram. Time-kill curves of colistin and plumbagin alone or their combination against colistin-resistant *Pseudomonas aeruginosa*. PLU, Plumbagin; COL, colistin.

As shown in Figure 1, the synergistic effect of plumbagin and colistin against the seven strains was further verified using the time-kill curve. The concentration of drug used for the time-kill curve was derived from the checkerboard analysis with FICI <0.5 plumbagin concentrations of 32 and 64 μg/ml, and colistin concentrations of 0.5, 1, and 2 μg/ml, respectively. The monotherapy group showed little or no time-dependent bactericidal activity compared to the control group. The combination of plumbagin and colistin significantly reduced the growth of COL-R PA beyond 3 log_10_ CFU/ml within 24 h. However, for TL1671, the combinatorial inhibition effect was weak after 12 h, as the bacteria started regressing then.

### The combination of colistin and plumbagin could inhibit biofilm formation and eradicate the mature biofilms

**FIGURE 2:**
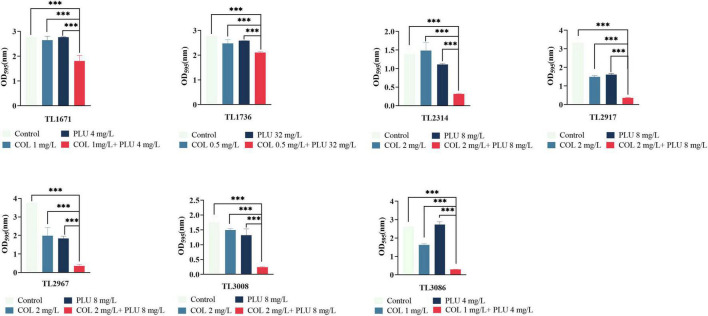
The combination of plumbagin and colistin inhibits biofilm formation of *Pseudomonas aeruginosa*. The selected drug concentration was derived from the checkerboard assay with fractional inhibitory concentration index (FICI <0.5). ***P < 0.001 significance were analyzed by One-way ANOVA. PLU, Plumbagin; COL, colistin.

**FIGURE 3:**
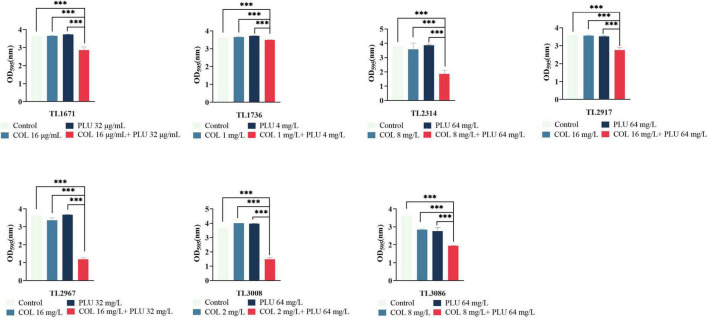
Eradication effect of plumbagin combined with colistin on *Pseudomonas aeruginosa* mature biofilm. The selected drug concentration was derived from the checkerboard assay with fractional inhibitory concentration index (FICI <0.5). ***P < 0.001 significance were analyzed by One-way ANOVA. PLU, Plumbagin and COL, colistin.

The effects of plumbagin and colistin, either alone or in combination, on the production of *P. aeruginosa* biofilms, were then investigated. The synergistic antimicrobial concentration against each strain was derived from the checkerboard assay. Compared to the monotherapy group and the control group, the combination of plumbagin and colistin decreased biofilm formation in all strains, as shown in Figure 2. Next, we explored whether plumbagin combined with colistin could remove the preformed biofilm. As shown in Figure 3, compared with the control and single-drug treatment groups, the plumbagin combined with colistin eradicated the biofilm of all bacterial strains.

In summary, these results showed that plumbagin combined with colistin could not only inhibit the formation of the biofilm of COL-R PA but, more importantly, eradicated the mature biofilm.

### The combination of colistin and plumbagin could inhibit biofilm formation and eradicate the mature biofilms

**FIGURE 4:**
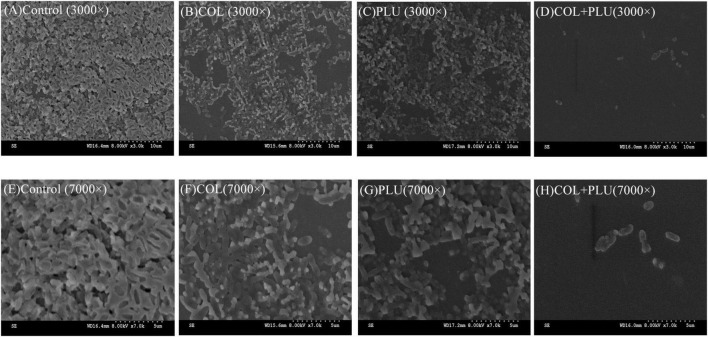
Scanning electron microscopy image of TL2314. **(A)** Luria Bertani (LB) broth control group, 3,000×; **(B)** colistin (1 μg/ml) single group, 3,000×; **(C)** plumbagin (16 μg/ml) single group, 3,000×; **(D)** plumbagin (16 μg/ml) combined with colistin (1 μg/ml) group, 3,000×; **(E)** LB broth control group, 7,000×; **(F)** colistin (1 μg/ml) single group, 7,000×; **(G)** plumbagin (16 μg/ml) single group, 7,000×; **(H)** plumbagin (16 μg/ml) combined with colistin (1 μg/ml) group, 7,000×.

Scanning electron microscopy was used to visualize the inhibition of plumbagin in combination with colistin on biofilm formation of COL-R PA. The results are shown in Figure 4. SEM images showed that the biofilms of the control group, the plumbagin monotherapy group (16 μg/ml), and the colistin monotherapy group (1 μg ml) were undamaged when observed at 3,000 × (Figures 4B,C) and 7,000 × (Figures 4F,G) magnifications, where the cells were characterized by complete morphology and high density, it even looked like a densely woven mesh. The combination group showed more significant changes compared to the control and monotherapy groups. It showed the structure of the biofilm was severely damaged, and the initially complete and compact biofilm was destroyed (Figures 4D,H). Cell morphology also changed, cells shrunk, and even cell debris appeared.

### At the experimental concentration, plumbagin had no significant effect on red blood cells

**FIGURE 5:**
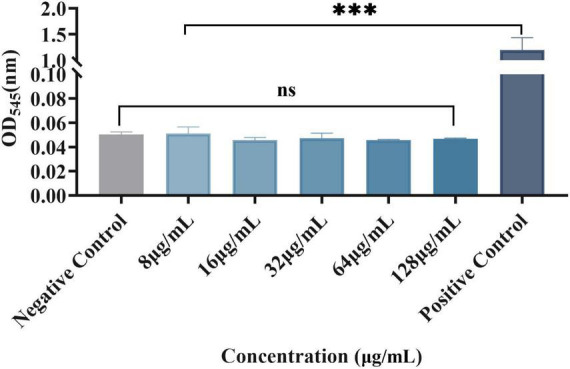
Effect of plumbagin on erythrocytes. Ns, not statistically significant; ***P < 0.001.

Erythrocytes were also used to evaluate plumbagin hemolysis, as shown in Figure 5. There were significant differences between the experimental group and the positive control, while there was no significant difference compared with the negative control. Therefore, at the experimental concentration, the RBCs did not show a significant hemolytic reaction to the plumbagin.

### Plumbagin combined with colistin could improve the survival rate of *Galleria mellonella in vivo*

**FIGURE 6:**
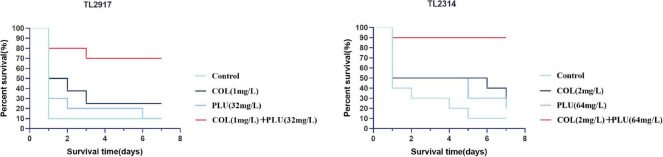
Survival rate of *Galleria mellonella* after different therapies. TL2314 and TL2917 as the experimental strains, and recorded the survival rate of *G. mellonella* over the 7-days observation period. PLU, plumbagin; COL, colistin.

The efficacy of plumbagin in combination with colistin was further validated *in vivo* by increasing the survival rate of the *G. mellonella* infection model. The results are shown in Figure 6. TL2917 and TL2314 were randomly selected as experimental strains. The survival rate of the TL2314-infected *G. mellonella* significantly reduced within 24 h in the control group, the plumbagin monotherapy group, and the colistin monotherapy group. In contrast, the combination of plumbagin (64 μg/ml) and colistin (2 μg/ml) maintained 90% survival after 168 h of treatment (*P* < 0.05) (Figure 6A). In the case of TL2917-associated infections, the survival rate of *G. mellonella* combined with colistin (1 μg/ml) and plumbagin (32 μg/ml) was higher than 50% (*P* < 0.05) (Figure 6B).

### Plumbagin combined with colistin plays an antibacterial role by increasing membrane permeability

**FIGURE 7:**
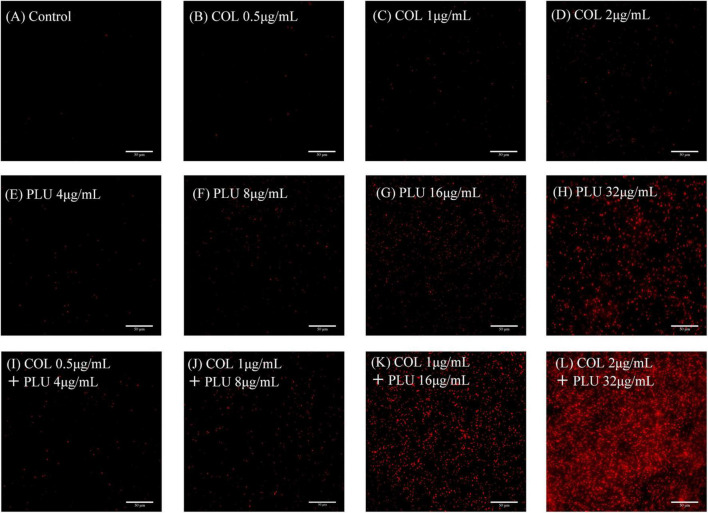
Membrane Permeability assay. TL2314 was selected for the test. **(A)** Luria Bertani (LB) broth control. **(B–D)** Cells were treated with colistin at 0.5 **(B)**, 1 **(C)**, 2, **(D)** μ g/ml. **(E–H)** Cells treated with plumbagin at 4 **(E)**, 8 **(F)**, 16 **(G)**, and 32 μ g/ml **(H)**. **(I–L)** Cells were exposed to the combination of plumbagin and colistin.

We evaluated the cell membrane permeability of TL2314 using PI. As revealed by fluorescence microscopy analysis in Figure 7, colistin alone had a minor effect on cell membrane permeability (Figures 7B–D). Preincubation of cells with plumbagin, on the other hand, resulted in a dose-dependent rise in fluorescence intensity due to PI absorption and DNA binding, indicating that the cell membrane’s integrity was steadily deteriorating (Figures 7E–H). And the combined groups were more significant than the monotherapy groups (Figures 7I–L).

## Discussion

*Pseudomonas aeruginosa* can infect immunocompromised patients and individuals with cystic fibrosis (Chatterjee et al., 2017). On the one hand, *P. aeruginosa* can form a variety of virulent factors, such as pyocyanin and elastase, harmful to human health (Yang et al., 2016); On the other hand, *P. aeruginosa* has a solid ability to form biofilms, which can colonize in a variety of environments and medical devices and is difficult to remove (Lee et al., 2020).

Colistin is an active agent against MDR gram-negative pathogens that frequently represents the mainspring of life-threatening infections, especially carbapenem-resistant *P. aeruginosa*, *A. baumannii*, *K. pneumoniae*, and *E. coli* (El-Sayed Ahmed et al., 2020). The number of GNB resistant to colistin has increased recently, posing many obstacles to treating colistin resistance. Whereas, developing and applying new antibacterial agents are costly and time-consuming. Nature is the primary source of biologically active substances. As stated by Fleming: “I did not invent penicillin. Nature did that. I only discovered it by accident” (Cappiello et al., 2020). As a result, natural compounds have been used as adjuvants for commonly used antibacterial drugs.

Naphthoquinone-producing plants have been exploited for centuries for their numerous pharmacological applications (Widhalm and Rhodes, 2016). Plumbagin is one of the substances, which is extracted from the plants of Plumbago. Research has pointed out that plumbagin can also inhibit virulence and biofilm-related QS of GNB (Qais et al., 2021). Besides, plumbagin combined with gentamicin has a synergistic effect on carbapenem-resistant *K. pneumoniae* (Chen et al., 2020). However, no studies combine plumbagin and colistin against ColR -*P. aeruginosa*.

In this study, we investigated plumbagin’s *in vitro* and *in vitro* effects combined with colistin against COL-R PA. Plumbagin combined with colistin displayed a substantial synergistic impact against COL-R PA, according to the checkerboard assay and time-kill curve. However, in the time-kill curve against TL1736, bacteria gradually grew after the drug combination treatment for 72 h, possibly attributed to either difference in the strains or the presence of persisters. However, the detailed mechanism for this is still under investigation in our laboratory.

In addition, the combination of the plumbagin and the colistin inhibits the biofilm’s formation and significantly eradicates the preformed biofilm. The image of SEM showed that compared with the monotherapy group, the bacterial burden in the plumbagin combined with the colistin group was significantly reduced, and the morphology was slightly changed. The results show that the combination of two drugs has great clinical significance, inhibiting biofilm formation in the early stage and removing biofilm in the late stage.

According to the erythrocytes hemolysis test, plumbagin had no significant effect on RBCs at the experimental concentration. Then, to study the synergistic effect of plumbagin and colistin *in vivo*, we experimented with an *in vivo* infection model of *G. mellonella*. The results showed that the survival rate of *G. mellonella* was significantly increased in the drug combination-treated group. In the past decades, colistin has been abandoned due to nephrotoxicity and neurotoxicity. The combination of plumbagin and colistin can significantly reduce the dosage of colistin, thus reducing the side effects caused by colistin. These results prove the viable potential of this combination *in vivo* in the future.

Based on the fluorescence microscope findings, the synergistic antibacterial mechanisms of plumbagin and colistin were also investigated. Our results showed that plumbagin alone could increase membrane permeability, and the combined group was more significant. Therefore, our drug combination can significantly increase the membrane permeability and yield bacteriostatic and bactericidal effects.

To our knowledge, plumbagin has shown substantial synergistic antibacterial activity with colistin against COL-R PA *in vitro* and *in vivo*. The novelty of our study lies in that the combination of plumbagin and colistin cannot only inhibit the formation of biofilm but also eradicate the mature biofilm. Also, plumbagin illustrated a negligible hemolysis effect. Collectively, this study may provide a new approach to COL-R PA infection.

## Conclusion

To our knowledge, this is the first study of synergistic activity of colistin in combination with plumbagin against COL-R PA. A series of experiments *in vitro* and *in vivo* have proved that plumbagin and colistin have synergistic effects on COL-R PA. Plumbagin in combination with colistin restores colistin activity *in vitro* and *in vivo*, which is encouraging. Our results have a potential to aid the clinical to treat infections caused by COL-R PA.

## Data availability statement

The original contributions presented in this study are included in the article/supplementary material, further inquiries can be directed to the corresponding authors.

## Author contributions

YW conducted the experiments, analyzed the data, and wrote the manuscript. JK, XZ, YaL, LY, and YZ participated in the experiments. ZH, JC, and LC participated in the analysis of the results. TZ and YoL helped design the study. All authors contributed to the article and approved the submitted version.
